# Supplementary material for: Point estimation for adaptive trial designs I: a methodological review
Source: Stat Med. Author manuscript; Available in PMC 2023 Jan 30. (PMC7613995; doi:10.1002/sim.9605)
Supplement: Supplementary file 1 [file EMS157773-supplement-Supplementary_File.pdf]

Methods of Estimation

3.1 Unbiased estimation

Paper title & authors	Trial context	Advantages / limitations	Code/software available?	Case studies?
Improved approximation for estimation following closed sequential tests<br><br>Kim (1988)<br><br>https://doi.org/10.1093/biomet/75.1.121	Focuses on estimating the mean of a normal distribution with known variance following a class of sequential tests studied by Woodroofe<br><br>Proposes modified MLE, median unbiased estimator, and midpoint of a 90% confidence interval	The MLE and its modification are very sensitive to the magnitude of the stopped random walk, while the median unbiased estimator and the midpoint of the 90% confidence interval are not and are thus robust<br><br>However modified MLE performs best in terms of bias and MSE	No	None
Two-stage conditionally unbiased estimators of the selected mean<br><br>Cohen & Sackrowitz (1989)<br><br>https://doi.org/10.1016/0167-7152(89)90133-8	Two-stage drop-the-loser trial where only the best treatment is taken forward to stage 2<br><br>Treatment outcome is normally distributed with known (or unknown) variance, or gamma distributed<br><br>Derives expression for UMVCUE	Gives simple analytical formula for UMVCUE<br><br>Low computational burden<br><br>Zero bias but high MSE (although conditional MSE is lower than MLE)	No	None

The bias of the sample proportion following a group sequential phase II clinical trial<br><br>Chang et al. (1989)<br><br>https://doi.org/10.1002/sim.4780080505	Investigates numerically the bias of the MLE of the binomial response probability, p, in group sequential phase II clinical trials and finds that its magnitude is less than 0.025 in all cases investigated<br>Applies Whitehead's idea to propose a bias-adjusted estimator that reduces the bias substantially and reduces the MSE as well in a certain range of p<br>Evaluates the UMVUE	If one does not mind a bias of 0.025, one may find the sample proportion a suitable estimator for p because of its simplicity and easy explanation<br>If one is concerned with bias, the bias-adjusted estimator may be a good choice<br>The UMVUE has a higher MSE than the sample proportion or the bias-adjusted estimator for most values of *p*	No	None
Point estimation following group sequential tests<br><br>Kim (1989)<br><br>https://www.jstor.org/stable/2531502	Considers three point estimators for a normal mean following group sequential tests: the MLE, MUE and the midpoint of an exact 90% confidence interval	MUE and midpoint estimator have a "great reduction in bias", but can have an increase in the MSE and variance. However, the reduction in bias is to a much greater degree than the increase in variance.<br><br>Midpoint estimator seems consistently better than MUE in terms of bias	No	None
Parameter estimation following group sequential hypothesis testing<br><br>Emerson and Fleming (1990)<br><br>https://www.jstor.org/stable/2337110	Investigates estimation following a group sequential hypothesis test for the mean of a normal distribution with known variance<br><br>Investigates the use of a median unbiased estimate based on the sample mean ordering	Compares MLE, median-unbiased estimates and UMVUE<br><br>The bias adjusted mean has the least absolute bias of the biased estimators and lowest mean squared error of all the estimators almost uniformly	No	None

Unbiased estimation of the parameter of a selected binomial population<br><br>Tappin (1992)<br><br>https://doi.org/10.1080/03610929208830831	Two-stage drop-the-loser trial where only the best treatment is taken forward to stage 2<br><br>Treatment outcome is binary<br><br>Derives expression for UMVUE	Gives simple analytical formula for UMVCUE<br><br>Low computational burden	No	None
Estimation after sequential testing: a simple approach for a truncated sequential probability ratio test<br><br>Woodroofe (1992)<br><br>https://doi.org/10.1093/biomet/79.2.347	Shows how to calculate the MUE for estimation a normal mean following a truncated sequential probability test	Proposed estimator is “median unbiased to a high order” but not exact	No	None
A computationally simpler algorithm for the UMVUE of a normal mean following a group sequential trial<br><br>Emerson and Kittelson (1997)<br><br>https://www.jstor.org/stable/2533122	Gives analytical expression for the UMVUE for a group sequential trial with normally distributed outcomes (and known variance)	Gives a computationally efficient algorithm to calculate the UMVUE exactly<br><br>Note no comparisons made with other estimators	No	None

Unbiased estimation following a group sequential test<br><br>Liu and Hall (1999)<br><br>https://academic.oup.com/biomet/article/86/1/71/255103	Derives technical conditions under which a UMVUE exists for a group sequential test with a Brownian motion	Gives analytical formula for UMVUE<br>Gives examples of alternative unbiased estimators that are “peculiar and unacceptable”, one having arbitrarily large variance	No	None
Conditional estimation following a group sequential clinical trial<br><br>Troendle and Yu (1999)<br><br>https://www.tandfonline.com/doi/abs/10.1080/03610929908832376	Gives three estimation methods to reduce the conditional bias in estimating the treatment difference for a group sequential trial with normally distributed outcomes, where the conditioning is on the stopping time	Shows through simulation that unconditionally unbiased estimators remain unbiased by overestimating the effect when there is early stopping, while underestimating the effect when the trial stops late<br><br>Proposed conditional estimators reduce the conditional bias, and can have similar conditional MSE to the usual MLE	No	None
Estimation of a secondary parameter in a group sequential clinical trial<br><br>Gorfine (2001)<br><br>https://onlinelibrary.wiley.com/doi/epdf/10.1111/j.0006-341X.2001.00589.x	Investigates estimation of secondary parameters in group sequential clinical trials: the primary parameter is the overall mean of a normally-distributed response variable, and the secondary parameter is the mean of this response in a sub-group of subjects.<br><br>Derives analytical expression for UMVUE for the secondary parameter and compares it with the naive estimator	For naive estimator, the sampling proportions of the subgroup have a crucial effect on the bias: as the sampling proportion of the subgroup at or just before the stopping time increases, the bias of the naive subgroup parameter estimator increases as well.<br><br>Proposes calculating UMVUE using Monte Carlo algorithm to avoid	No	Beta-Blocker Heart Attack Trial (a group sequential clinical trial)

		multidimensional integration - could be computationally intensive<br><br>Differences in MSE between the UMVUE and naive estimator are negligible.		
Flexible two-stage designs: An overview<br><br>Bauer et al. (2001)	Sketches construction of median unbiased estimators in flexible two-stage designs (based on combination tests and conditional error functions)	[Could not access paper]	?	?
Recursive combination tests<br><br>Brannath et al. (2002)<br><br>https://www.tandfonline.com/doi/abs/10.1198/016214502753479374	Gives general method to calculate median unbiased point estimates when using recursive combination tests for multi-stage adaptive trials	Allows for "simple computation" of median unbiased point estimates<br><br>No comparisons given for MSE	No	None
Unbiased estimation of secondary parameters following a sequential test<br><br>Liu and Hall (2001)<br><br>https://www.jstor.org/stable/267	Proposes UMVUE for a correlated secondary Gaussian process,	No simulation results given or comparison made with other estimators	No	None

3457				
Sequential tests and estimators after overrunning based on maximum-likelihood ordering<br><br>Hall and Liu (2002)<br><br>https://academic.oup.com/biomet/article/89/3/699/252219	Shows how to calculate a median unbiased estimator for a sequential trial (under Brownian motion) after overrunning	Median unbiased estimate needs to be obtained numerically<br><br>No comparisons given for MSE	No	Multicenter Automatic Defibrillator Implantation Trial (MADIT), which is a group sequential trial
A unified theory of two-stage adaptive designs<br><br>Liu et al. (2002)<br><br>https://www.tandfonline.com/doi/abs/10.1198/016214502388618852	Gives technical conditions under which estimators are unbiased, UMVUE and consistent for general two-stage adaptive designs	Theoretical results that need to be applied to a specific trial context	No	None
Confidence intervals following group sequential tests in clinical trials with multivariate observations<br><br>Lee et al. (2002)<br><br>https://doi.org/10.1080/00949650212386	Proposes an exact confidence interval for parameters of interest in a group sequential trial with repeated measures	Not about point estimation, but could use CIs to construct a MUE	No	Hypothetical example based on study of calcium supplements on bone density

Inference about a secondary process following a sequential trial<br><br>Hall and Yakir (2003)<br><br>https://academic.oup.com/biomet/article/90/3/597/231502	Constructs conditional median-unbiased estimators for a secondary parameter after a group-sequential or fully-sequential test	Proves that the proposed median-unbiased estimator is uniformly most accurate (conditionally) unbiased<br><br>Calculating the proposed median-unbiased estimator is complicated, and requires a Monte Carlo procedure<br><br>No comparisons given for MSE	No	Multicenter Automatic Defibrillator Implantation Trial (MADIT), which is a group sequential trial
Estimation and Confidence Intervals after Adjusting the Maximum Information<br><br>Lawrence and Hung (2003)<br><br>https://doi.org/10.1002/bimj.200390001	Focuses on an adaptive test procedure for testing for a treatment effect lower than expected that results in a sample size adjustment based on the observed sample path at an interim time of the trial.<br><br>Proposes MUE (midpoint of a confidence interval)	Proposed MUE has uniformly smaller bias than the naive estimator<br><br>When the true difference is much smaller than the initial guess, then the naive estimator is a more efficient estimator as measured by the MSE; otherwise the MSE are “roughly equivalent”	No	Hypothetical example based on trial testing the effect of a new drug for the prevention of myocardial infarction
On the estimation of the binomial probability in multistage clinical trials<br><br>Jung and Kim (2004)<br><br>https://doi.org/10.1002/sim.1653	Multistage sequential design with binary responses<br><br>Derives analytical formula for the UMVUE	In two-stage setting, the MLE has smaller MSE for smaller p-values than UMVUE, but larger MSE for larger p-values. There appears to be some efficiency loss with the UMVUE as compared to the MLE, particularly for optimal designs, a reasonable price for unbiasedness<br><br>Bias-adjusted MLE can have larger MSE than UMVUE in some situations	No	None

Supplementary analysis of probabilities at the termination of a group sequential phase II trial<br><br>Liu et al. (2005)<br><br>https://doi.org/10.1002/sim.1990	Considers estimation of various probabilities after termination of a group sequential phase II trial<br>Shows that the conventional MLE (sample proportion) is biased<br>Proposes two alternative estimators to correct for bias, a bias-reduced estimator obtained by using Whitehead's bias-adjusted approach, and an unbiased estimator from the Rao-Blackwell method of conditioning	All three estimation procedures are shown to have certain invariance properties in bias<br>Estimators of a probability and their bias and precision can be evaluated through the observed response rate and the stage at which the trial stops, thus avoiding extensive computation	No	None
Estimation following a group sequential test for distributions in the one-parameter exponential family<br><br>Liu et al. (2006)<br><br>http://www3.stat.sinica.edu.tw/statistica/oldpdf/A16n110.pdf	Considers unbiased estimation following a group sequential test for distributions in a one-parameter exponential family<br><br>Derives unbiased Rao-Blackwell estimator, which is UMVUE if completeness holds	Gives analytical expressions for Rao-Blackwell estimator<br><br>Computation of the unbiased estimators can be complex and extensive, especially when the number of looks, is relatively large (≥ 4)<br><br>No comparisons made with other estimators	No	None
On design and inference for two-stage adaptive clinical trials with dependent data<br><br>Liu and Pledger (2006)	Considers general two-stage adaptive designs for clinical trials where data from the two stages are dependent, e.g where the second stage includes additional follow-up data of the first stage patients	Theoretical results that need to be applied to a specific trial context	No	None

https://doi.org/10.1016/j.jspi.2005.08.015	Provides a method for obtaining unbiased estimators for “construct parameters” (those that are invariant under a general adaptation rule)			
Planning and analyzing adaptive group sequential survival trials<br><br>Wassmer (2006)<br><br>https://doi.org/10.1002/bimj.200510190	Shows how to calculate median unbiased estimates for adaptive group sequential survival trials<br><br>MUEs based on either RCIs or final analysis CIs	Unadjusted estimator has higher bias for O’Brien Fleming than for Pocock, whereas MUE has similar bias for both	ADDPLAN	NSCLC Trial, which is a three-stage group sequential trial
Estimation in flexible two stage designs<br><br>Brannath et al. (2006)<br><br>https://doi.org/10.1002/sim.2258	Gives an overview of point and interval estimates for flexible two stage designs, focusing on sample size (re)assessment rules	Shows that the absolute mean bias of the MLE is bounded, and is at most 40% of the sd of the first stage mean. In addition, the maximum mean bias in a flexible two stage design is in general not larger than in a conventional group sequential design.<br><br>Simulations indicate that the mean bias of median unbiased and MLE is usually small compared to their MSE, and that the mean unbiased estimate may perform badly in terms of the MSE. Also shows median unbiased estimator can have similar mean bias to the MLE.	No	Trial on reperfusion therapy for acute myocardial infarction, which was a two-stage trial with sample size reassessment

p-Value Calculation for Multistage Phase II Cancer Clinical Trials<br><br>Jung et al. (2006)<br><br>https://doi.org/10.1080/10543400600825645	Investigates some approaches to p-value calculation in analyzing multi-stage Phase II clinical trials that have a binary variable<br><br>Note focus is more on p-values rather than bias	Whatever estimator is used, the p-values depend on the ordering, but not on the estimates. So, two estimators will result in exactly the same p-value if they have the same ordering. Chang et al. (1989) correct the bias of the MLE using Whitehead's (1986) approach. The bias-corrected estimator has exactly the same ordering as the MLE, so the two estimators result in the same p-value.<br><br>Further, both MLE and UMVUE increase in s for each m. Hence, the two estimators will have the same ordering as long as their ordering matches at the boundaries. Otherwise can have discordant p-values<br><br>Only the UMVUE ordering provides p-values with desirable properties	No	None
Completeness and unbiased estimation of mean vector in the multivariate group sequential case<br><br>Liu et al. (2007)<br><br>https://doi.org/10.1016/j.jmva.2006.01.001	Considers estimation following a multivariate group sequential test, i.e. with random samples from a multivariate normal.<br><br>Presents a Rao–Blackwell type unbiased estimator, which is UMVUE among truncation-adaptable statistics	Gives a recursive formula for Rao-Blackwell estimator. Numerical computation is time-consuming.<br><br>No comparisons made between estimators	No	None

Proper inference from Simon's two-stage designs<br><br>Koyama and Chen (2007)<br><br>https://doi.org/10.1002/sim.3123	Shows how to report p-values, point estimates and CIs for Simon's two-stage designs, including when stage 2 sample size is different from the one planned<br>Proposes MUE (p-value = 0.5)	No comparisons between estimators given	Link to website with software does not seem to work	None
Point and interval estimation of accuracies of a binary medical diagnostic test following group sequential testing<br><br>Shu et al. (2008)<br><br>https://doi.org/10.1098/rsta.2008.0041	Compares the bias and mean squared errors of the MLE and Rao–Blackwell unbiased estimators for the sensitivity and specificity of a binary medical diagnostic test following a group sequential procedure	Derives a recursive expression for the Rao–Blackwell estimator<br><br>Simulations indicate that the MLE (slightly) underestimates both sensitivity and specificity. The MSE of the MLE of sensitivity decreases more than that of the Rao–Blackwell estimator when specificity increases, but “the magnitude of the reduction is not appealing taking into account the bias of the MLE”	No	None
Unbiased estimation of selected treatment means in two-stage trials<br><br>Bowden and Glimm (2008)<br><br>https://doi.org/10.1002/bimj.200810442	Two-stage drop-the-loser trial where treatment outcomes are normally distributed with known variances<br><br>Extends Cohen and Sackrowitz (1989) to calculate UMVCUE for unequal stage 1 and 2 sample sizes, and for when the quantity of interest is the best, second best, or j-th best treatment out of k	Gives relatively simple analytical expression for UMVCUE, with low computational burden<br><br>MLE can have fairly substantial bias for small sample sizes<br><br>MSE of UMVCUE increases dramatically as the amount of information (at the	No	None

		interim analysis) increases for the UMVCUE, but MSE is stable for the MLE<br><br>In terms of MSE, the UMVCUE generally performs worse when used to estimate a treatment from the middle of the ordered range<br><br>*Conditional* MSE of MLE can increase dramatically if the wrong selection is made at interim. This tendency is much reduced with the UMVCUE		
Point and Interval Estimation of Primary and Secondary Parameters in a Two-Stage Adaptive Clinical Trial<br><br>Liu et al. (2008)<br><br>https://doi.org/10.1080/10543400701697125	Considers a two-stage adaptive trial with normally-distributed observations. At the interim analysis a new sample size is calculated based on the primary endpoint of interest.<br><br>For the primary endpoint, compares the MLE, Whitehead's bias-adjusted approach, an unbiased Rao-blackwellized estimator, and two weighted estimators<br><br>For the secondary endpoint, compares a bias-adjusted estimate and an unbiased Rao-blackwellized estimator	For the primary endpoint, the bias-adjusted estimate always has smaller bias than the other estimators (apart from the unbiased estimator) whereas the maximum likelihood estimate has the largest<br><br>The unbiased estimator tends to have larger MSE, whereas the others have comparable MSE.<br><br>Overall, the bias-adjusted estimator tends to be the best among the estimators considered	No	None

Conditional estimation of sensitivity and specificity from a phase 2 biomarker study allowing Early termination for futility<br><br>Pepe et al. (2009)<br><br>https://doi.org/10.1002/sim.3506	Two-stage design testing the sensitivity of dichotomous biomarker, with early stopping for futility<br><br>Derives and compares the UMVUE, UMVCUE, median-adjusted estimator and mean-adjusted estimator	Naive estimator can have “substantial” upward bias<br><br>UMVCUE is unbiased and, when early stopping is unlikely, its precision is comparable with the naive estimator<br><br>Mean- and median-adjusted estimators have similar performance to UMVCUE, but their computation is more difficult<br><br>UMVUE can be conditionally biased	No	None
On Bayesian estimators in multistage binomial designs<br><br>Bunouf and Lecoutre (2008)<br><br>https://doi.org/10.1016/j.jspi.2008.02.014	Considers a new class of Bayesian estimators for a proportion in multistage binomial designs<br><br>Compares new Bayesian estimators to Whitehead’s and UMVUE	Exact calculations of posterior mode/mean can be computationally intensive<br><br>For two-stage designs, easy-to-use approximation of the posterior mode is given<br><br>Compared to MLE, the advantage of the Bayesian estimators and Whitehead estimator is substantial. In compensation for its unbiasedness, UMVUE exhibits less advantageous characteristics in terms of relative efficiency<br><br>In setting of planning experiments to assess the probability of rare events,	No	None

		Bayesian estimators have small bias and strongly increasing relative efficient compared to MLE		
Point and Interval Estimation of Primary and Secondary Parameters in a Two-Stage Adaptive Clinical Trial<br><br>Liu et al. (2008)<br><br>https://doi.org/10.1080/10543400701697125	Considers a two-stage adaptive trial with normally-distributed observations. At the interim analysis a new sample size is calculated based on the primary endpoint of interest.<br><br>For the primary endpoint, compares the MLE, Whitehead's bias-adjusted approach, an unbiased Rao-blackwellized estimator, and two weighted estimators<br><br>For the secondary endpoint, compares a bias-adjusted estimate and an unbiased Rao-blackwellized estimator	For the primary endpoint, the bias-adjusted estimate always has smaller bias than the other estimators (apart from the unbiased estimator) whereas the maximum likelihood estimate has the largest<br><br>The unbiased estimator tends to have larger MSE, whereas the others have comparable MSE.<br><br>Overall, the bias-adjusted estimator tends to be the best among the estimators considered	No	None
Exact confidence bounds following adaptive group sequential tests<br><br>Brannath et al. (2009)<br><br>https://doi.org/10.1111/j.1541-0420.2008.01101.x	Shows how to obtain median-unbiased estimators following adaptive group sequential tests. These MUEs can be based on the stage-wise adjusted CIs or RCIs.	Stage-wise adjusted CIs produces median unbiased point estimates, but the RCI can have negative bias.	No	Hypothetical example based on a deep brain stimulation trial for Parkinson's disease

Adaptive designs for confirmatory clinical trials<br><br>Bretz et al. (2009)<br><br>https://doi.org/10.1002/sim.3538	Point estimation section focuses on sample size reestimation for a two-stage trial	Recaps results of Brannath et al. (2006) and Bowden and Glimm (2008)	No	None
Estimation and Confidence Intervals for Two-Stage Sample-Size-Flexible Design with LSW Likelihood Approach<br><br>Wang et al. (2010)<br><br>https://link.springer.com/article/10.1007%2Fs12561-010-9023-0	Describes a method for the point and confidence interval estimation for the likelihood approach of sample size adaptive design proposed by Li et al. (LSW likelihood method).<br><br>The point estimator is a median unbiased estimator	The median unbiased estimate is nearly (mean) unbiased in all cases, and has smaller RMSE than the naïve estimate in most cases except when the true delta is close to the null case	No	Case study using coronary artery disease trial
Parameter estimation following an adaptive treatment selection trial design<br><br>Luo et al. (2010)<br><br>https://doi.org/10.1002/bimj.200900134	Two-stage drop-the-losers trial, where number of responses follow a Binomial distribution<br><br>Derives bias-adjusted MLE based on method of moments, and compares this with naive estimator and Rao-Blackwell estimator	Bias-adjusted MLE has to be calculated numerically<br><br>Simulations suggest that the bias-adjusted MLE has relatively low RMSE and acceptably small bias – generally smaller than that of the naive estimator and practically comparable with the Rao–Blackwell estimator. Its MSE	No	Hypothetical example based on study in colorectal cancer

		performed a bit better than that of the Rao–Blackwell-type estimator. In addition, it can be applied for the one-stage situation		
Likelihood inference for a two-stage design with treatment selection<br><br>Bebu et al. (2010)<br><br>https://doi.org/10.1002/bimj.200900170	A conditional likelihood-based approach is proposed for the parameters of interest in a two-stage design with treatment selection after the first stage and normally distributed responses.<br><br>Compares conditional MLE (cMLE) with naive MLE and UMVCUE	The bias of the cMLE estimator is also reasonably small, but always negative. On the other hand, the naive MLE has a larger bias that is always positive<br><br>However naive MLE has smallest MSE	No	None
On efficient two-stage adaptive designs for clinical trials with sample size adjustment<br><br>Liu et al. (2012)<br><br>https://doi.org/10.1080/10543406.2012.678226	Proposes a likelihood-based two-stage adaptive design, where sample size adjustment is derived from a group sequential design using cumulative conditional power<br><br>Provides methods for median unbiased and minimum variance unbiased estimates	Median unbiased estimator has a smaller MSE, but minimum variance unbiased estimate has a smaller bias. “It does not appear compelling that one estimator outperforms the other”	No	None
Stopping a trial early - and then what?	Discusses methods for correcting the bias in observed effect sizes, confidence intervals, and p-values for trials stopped	In RALES, the effect of not correcting for bias is negligible	No	Randomized Aldactone Evaluation Study (RALES)

Wittes (2012)<br><br>https://doi.org/10.1177%2F1740774512454600	early and to show the extent to which such correction would have modified the conclusions of the Randomized Aldactone Evaluation Study (RALES)<br><br>Uses median-unbaised estimator based on midpoint of CIs with stagewise ordering			
Conditional estimation after a two-stage diagnostic biomarker study that allows early termination for futility<br><br>Koopmeiners et al. (2012)<br><br>https://doi.org/10.1002/sim.4430	Discuss conditional estimation of parameters of interest after a two-stage study that allows early termination for futility<br><br>Compares conditional mean adjusted MLE, conditional median adjusted MLE and the UMVCUE	Mean and median adjusted estimators have similar standard errors to UMVCUE<br><br>MLE has the smallest standard error when true parameter is small and there is a high probability of termination, but there is little difference when true parameter is larger and early termination is rare	No	None
What inference for two-stage phase II trials?<br><br>Porcher and Desseaux (2012)<br><br>https://bmcmedresmethodol.biomedcentral.com/articles/10.1186/1471-2288-12-117	Compares different approaches for point and confidence intervals estimation for Simon's two-stage design	For point estimation, the UMVUE was unbiased both when the actual number of patients recruited was equal to or differed from the pre-planned value. The bias corrected estimator had negligible bias and slightly lower RMSE than the UMVUE only when the true response rate was close to its value under the null hypothesis. "Both estimators performed better than the others and can thus be recommended"	No	None

Estimation of secondary endpoints in two-stage phase II oncology trials<br><br>Kunz and Kieser (2012)<br><br>https://doi.org/10.1002/sim.5585	Presents UMVUEs for secondary endpoints in two-stage designs that allow stopping for futility and efficacy<br><br>Compares the MSE of the UMVUE and the MLE and investigate the efficiency of the UMVUE.	Analytical formula given for UMVUE<br><br>Comparing the MSEs of the UMVUE and the MLE shows that none of the estimators is in general superior to the other in terms of MSE. In general, the MSEs of the UMVUE and the MLE are very close to each other, and depending on the true values, the MSE of one or the other is slightly smaller.	No	Phase II trial of OSI-7904L in patients with adenocarcinoma
Adaptive designs for noninferiority trials<br><br>Gao et al. (2013)<br><br>https://doi.org/10.1002/bimj.201200034	Presents a method of testing for noninferiority followed by testing for superiority in a general adaptive group sequential trial<br><br>Obtains median unbiased point estimate for the efficacy parameter	Calculating median unbiased estimate can be computationally intensive<br><br>No comparisons made with other estimators	No	None
Exact inference for adaptive group sequential designs<br><br>Gao et al. (2013)<br><br>https://doi.org/10.1002/sim.5847	Provides a method for obtaining median-unbiased point estimates and exact two-sided confidence intervals for adaptive group sequential designs	Calculating median unbiased estimate can be computationally intensive	No	Hypothetical example based on deep brain stimulation trial for Parkinson's disease

Conditionally unbiased estimation in phase II/III clinical trials with early stopping for futility<br><br>Kimani et al. (2013)<br><br>https://doi.org/10.1002/sim.5757	Seamless phase II/III clinical trials with normally distributed endpoint<br><br>Derives unbiased estimator based on Rao-Blackwellisation, and a bias-adjusted estimator	The bias-adjusted estimator overcorrects for bias, and the overcorrection increases with selection time but decreases as the value of the futility boundary increases. The naive estimator has the lowest MSE at all selection times for all scenarios. The unbiased estimator and the bias-adjusted estimator have approximately equal MSE	No	Case study based on a comparison of three doses of an experimental drug for generalized anxiety disorder with a placebo
An evaluation of inferential procedures for adaptive clinical trial designs with pre-specified rules for modifying the sample size<br><br>Levin et al. (2014)<br><br>https://doi.org/10.1111/biom.12168	Extends group sequential orderings of the outcome space based on the stage at stopping, likelihood ratio statistic, and sample mean to the adaptive setting in order to compute median-unbiased point estimates and exact CIs	The bias adjusted mean demonstrates the lowest MSE among candidate point estimates<br>A conditional error-based approach in the literature has the benefit of being the only method that accommodates unplanned adaptations	Yes (R code)	None
Conditionally unbiased and near unbiased estimation of the selected treatment mean for multistage drop-the-losers trials<br><br>Bowden and Glimm (2014)	Derive unbiased and near-unbiased estimates for the multistage drop-the-losers design	Unbiased estimator has a large MSE<br><br>Conditional MLE computationally intensive. Overcorrects for bias but has reduced MSE compared with unbiased estimator	Yes (R code)	None

https://dx.doi.org/10.1002%2Fbimj.201200245	Unbiased estimator based on Rao-Blackwellisation, and near-unbiased estimator based on conditional MLE			
Correcting for bias in the selection and validation of informative diagnostic tests<br><br>Robertson et al. (2015)<br><br>https://doi.org/10.1002/sim.6413	Two-stage drop-the-loser trial for a binary classifier, with early stopping for futility<br><br>Derives analytical expression for the UMVCUE	Formula for UMVCUE is simple and easy to compute<br><br>UMVCUE is unbiased but often has a higher MSE than the MLE<br><br>Median-unbiased estimator showed no gain over the UMVCUE it terms of MSE	No	Family history screening tool validation study
Statistical inference for extended or shortened phase II studies based on Simon's two-stage designs<br><br>Zhao et al. (2015)<br><br>https://bmcmedresmethodol.biomedcentral.com/articles/10.1186/s12874-015-0039-5	Considers an inference method based on the likelihood ratio for Simon's two-stage design<br><br>Dervies conditional likelihood UMVUE	The conditional likelihood UMVUE has uniformly smaller biases than the estimate based on Koyama and Chen, especially when the underlying true probability is large.	No	GI06-101 trial in hepatobiliary cancer
Estimation after subpopulation selection in adaptive seamless trials	Derives the UMVCUE for two-stage adaptive seamless designs, for both	Compares MLE with UMVCUE. The MLE can be 'substantially biased, but has lower MSE	No	No (hypothetical trial for depression)

Kimani et al. (2015)<br><br>https://doi.org/10.1002/sim.6506	known and unknown subpopulation prevalences	The recommendation is to use the UMVCUE when the subpopulation is selected, but otherwise to use the MLE		
Unbiased estimation in seamless phase II/III trials with unequal treatment effect variances and hypothesis-driven selection rules<br><br>Robertson et al. (2016)<br><br>https://doi.org/10.1002/sim.6974	Seamless phase II/III trials for normally-distributed responses with unequal variances.<br><br>Derives analytical formula for the UMVUCE	MLE can have substantial bias<br><br>UMVCUE is unbiased, but has higher MSE than the MLE.	No	Case study based on trial comparing three experimental drugs with placebo for the treatment of anxiety disorder
Point estimation and p-values in phase II adaptive two-stage designs with a binary endpoint<br><br>Kunzmann and Kieser (2016)<br><br>https://doi.org/10.1002/sim.7200	Adaptive two-stage single-arm trial with a binary endpoint. The stage 2 sample size is determined based on the results of stage 1.<br><br>Derives a Rao-Blackwellised estimator<br><br>Proposes an optimal compatible (OC) estimator, which minimises the MSE subject to compatibility with the decision rule and monotonicity conditions	None of the point estimators previously known from the literature guarantees compatibility with the test decision<br><br>OC reduces MSE of MLE except for very small or very large values of the success probability<br><br>Rao-Blackwellised estimator is unbiased but has the highest MSE	No	None

		OC reduces absolute bias compared with MLE except for small values of the success probability, where is has a substantial positive bias		
Comparison of conditional bias-adjusted estimators for interim analysis in clinical trials with survival data<br><br>Shimura et al. (2017)<br><br>https://doi.org/10.1002/sim.7258	Reviews the characteristics of the conditional mean-adjusted estimator (CMAE), conditional median-adjusted estimator, conditional uniformly minimum variance unbiased estimator (CUMVUE), and weighted estimator and their CIs and compares their conditional bias, overall bias, and conditional MSE in clinical trials with survival endpoints	The CMAE reduces conditional bias and shows relatively small conditional MSE in trials terminated at the interim analysis<br>The CUMVUE has less bias and an acceptable conditional coverage probability in trials not terminated early	No	Breast cancer trial (CLEOPATRA)
Conditional estimation in two-stage adaptive designs<br><br>Broberg & Miller (2017)<br><br>https://doi.org/10.1111/biom.12642	Considers a design which permits raising the sample size when interim results look rather promising, and which retains the originally planned sample size when results look very promising<br>Compares the unconditional MLE, the conditionally unbiased Rao-Blackwell estimator (UMVCUE), the conditional median-unbiased estimator, and the conditional MLE with and without bias correction	The MLE performs well in the simulations in terms of MSE. However, it MLE does not possess any optimality features in the conditional inference setting.<br>The difference between the four conditional estimators was quite small in the scenarios considered. This was also reflected in terms of their bias (they had little or no bias) and their variance, which was similar.<br>The conditional Rao–Blackwell estimator has the advantage that it is unbiased in construction and has an explicit representation making computation simpler.	No	Schizophrenia trial

Unbiased estimation for response adaptive clinical trials<br><br>Bowden & Trippa (2017)<br><br>https://doi.org/10.1177/0962280215597716	Investigates the bias induced in the MLE of a response probability parameter, p, for binary outcome by the process of adaptive randomisation<br>Obtains a simple unbiased estimator for p<br>Explores two approaches to improve its precision based on inverse probability weighting and Rao–Blackwellisation	The bias of the MLE is small in magnitude and, under mild assumptions, can only be negative (causing one's estimate to be closer to zero on average than the truth)<br>The unbiased estimator has a large MSE<br>Approaches to improve MSE of unbiased estimator can be very computationally intensive	No	Glioblastoma trial with multiple experimental treatments
Point estimation in adaptive enrichment designs<br><br>Kunzmann et al. (2017)<br><br>https://doi.org/10.1002/sim.7412	Two-stage adaptive enrichment design where a binary biomarker is applied to select the patient population investigated in stage 2. The outcomes are assumed to be normally distributed with known variance.<br><br>Proposes alternatives to the MLE:<br><br>1. Empirical Bayes Estimator<br>2. Parametric Bootstrap Estimator<br>3. Conditional Moment Estimator<br>4. Hybrid estimator (UMVCUE and MLE or CME)	EBE and PBE exhibit a higher bias than the MLE in many situations<br><br>CME and hybrid consistently reduce the bias but there is a price to be paid in terms of MSE	No	Illustrative example based on MILLY phase II study in asthma
Interval and point estimation in adaptive Phase II trials with binary endpoint<br><br>Nhacolo and Brannath (2018)	Adaptive single-arm two-stage group sequential designs with a binary endpoint<br><br>Proposes point and interval estimation for adaptive designs in which the second stage sample size is a pre-specified	Proposed methods outperform the MLE (in terms of bias and MLE) when the true response probability is in the neighbourhood of values that are equal to or greater than the response probability under the alternative hypothesis	Code available upon request	None

https://doi.org/10.1177%2F0962280218781411	function of the first stage's number of responses. Approach is based on sample space orderings (i.e. median unbiased estimator)	Proposed methods can have noticeable negative bias however for other values of the true response probability		
Conditionally unbiased estimation in the normal setting with unknown variances<br><br>Robertson and Glimm (2018)<br><br>https://doi.org/10.1080/03610926.2017.1417429	Two-stage trials with treatment selection, where responses are normally distributed with unknown variance<br><br>Derives analytical expression for UMVCUE	MLE can have substantial positive bias<br><br>UMVUCE is unbiased but has higher MSE than MLE	No	INHANCE study
Uniformly minimum variance conditionally unbiased estimation in multi-arm multi-stage clinical trials<br><br>Stallard and Kimani (2018)<br><br>https://doi.org/10.1093/biomet/asy004	Multi-arm multi-stage clinical trials with treatment selection and early stopping for futility<br><br>Obtains UMVCUE conditional on selection with any specified rule or stopping for futility	The naive estimator is conditionally biased, overestimating the true effect. The bias is relatively small, but in some cases it is close to the difference in clinical response rates considered important in the trial design<br><br>MSE of UMVCUE and MSE are similar<br><br>"In a fully flexible approach, it is not clear that it is even possible to define the bias,	No	ADVENT trial

		as this would be an expectation over an unspecified sample space.”		
Exact Inference for Adaptive Group Sequential Designs<br><br>Mehta et al. (2019)<br><br>https://link.springer.com/chapter/10.1007%2F978-3-319-67386-8_10	Provides a method for obtaining median-unbiased point estimates for adaptive group sequential trials (i.e. those that permit sample size reestimation, alterations to the number and spacing of the interim looks, and changes to the error spending function based on an unblinded look at the accruing data)<br><br>The median-unbiased estimate is calculated by inverting one-sided p-values	Simulation results show negligible mean bias of the median-unbiased estimator<br><br>States that the estimator can be ‘easily computed’	No	None
Point estimation following two-stage adaptive threshold enrichment clinical trials<br><br>Kimani et al. (2018)<br><br>https://doi.org/10.1002/sim.7831	Develops point estimators for clinical trials that use the two-stage adaptive enrichment threshold design. The design consists of two stages, where in stage 1, patients are recruited in the full population. Stage 1 outcome data are then used to perform interim analysis to decide whether the trial continues to stage 2 with the full population or a subpopulation. The subpopulation is defined based on one of the candidate threshold values of a predictive biomarker.	Depending on the scenario, the bias of the naive estimator of the treatment effect in the selected subpopulation is substantial and can be negative or positive.<br><br>Recommends use of the UMVCUE - although it has larger MSE than some estimators, the bias eradicated in most estimators, the bias eradicated in most cases was larger than the difference in RMSEs<br><br>Since none of the estimators dominated in all simulation scenarios based on both	No	None

	Derives unbiased estimators (including UMVCUE), shrinkage estimators and bias-adjusted estimators	bias and MSE, an alternative strategy would be to use a hybrid estimator where the estimator used depends on the subpopulation selected. This would require a simulation study of plausible scenarios before the trial.		
Estimation of treatment effects following a sequential trial of multiple treatments<br><br>Whitehead et al. (2020)<br><br>https://doi.org/10.1002/sim.8497	Paper motivated by a trial in which four treatments for sepsis are to be compared, with interim analyses allowing the dropping of treatments or termination of the trial to declare a single winner or to conclude that there is little difference between the treatments that remain.<br><br>Estimation approach based on the method of Rao-Blackwellisation. Analytic approaches to determine such expectations are difficult and specific to the details of the design, and instead “reverse simulations" are conducted to construct replicate realisations of the first interim analysis from the final test statistics.	Reverse simulation approach can be computationally intensive<br><br>The remaining bias is small<br><br>In the two-treatment context, methods based on orderings of the final sample space are just as good for computing point estimates<br><br>The utility of the approach described here is in more complicated designs comparing multiple treatments or with flexible adaptive features, as reverse simulation is based only on the form of the stopping rules implemented and not on their theoretical properties	Yes (SAS)	Hypothetical data based on sepsis trial
Point and interval estimation in two-stage adaptive designs with	Considers estimation in two-stage adaptive designs that in stage 1 recruit	(Approximate) asymptotic UMVCUE has only small residual bias. In contrast, the	Yes (R code)	Constructs a hypothetical two-stage

time to event data and biomarker-driven subpopulation selection<br><br>Kimani et al. (2020)<br><br>https://doi.org/10.1002/sim.8557	patients from the full population. In stage 2, patient recruitment is restricted to the part of the population, which, based on stage 1 data, benefits from the experimental treatment.<br><br>For time-to-event data, derives a new asymptotically unbiased estimator (UMVCUE) for the log hazard ratio (as well as interval estimators), which is appropriate for several selection rules that are based on a single or multiple biomarkers, which can be categorical or continuous	naive point estimator can have substantial bias.<br><br>Unlike the case of normally distributed outcomes, compared with the naive estimator, the UMVCUE did not have markedly higher RMSE and in some simulation scenarios, it outperformed the naive estimator in terms of RMSE.<br><br>Authors expect the recommendation that the approximate UMVCUE is the best estimator to hold in most setting		enrichment trial using data from a single-stage trial that compared intravenous methotrexate (C-MTX) and high-dose methotrexate (HDMTX) in the treatment of T-cell acute lymphoblastic leukemia (T-ALL) in children
A comparison of estimation methods adjusting for selection bias in adaptive enrichment designs with time-to-event endpoints<br><br>Stefano et al. (2022)<br><br>https://doi.org/10.1002/sim.9327	Compares side-by-side the performances of six estimators of the treatment effects for two-stage enrichment ADs with time-to-event data:<br><br>● Naive estimator (MLE)<br>● UMVCUE<br>● Shrinkage estimators<br>● Bias-adjusted estimators<br><br>Focus is on conditional estimation (conditional on the selection made for the subgroups)	The naive estimator has the highest bias but very low variance, resulting in a moderate MSE compared to the other estimators.<br><br>The UMVCUE has a small constant positive bias (which is not zero because of the correlation bias, but is reduced by increasing the number of sub-populations) and the highest variance, resulting in the highest MSE.<br><br>The single-iteration bias-adjusted estimator has small bias, but more variance with respect to naive estimator, resulting in comparable MSE with respect to the naive estimator.	Yes (R code)	Case study based on a real trial in heart failure. The initial study was a group sequential design (without population selection). After the analysis, some subgroups with different efficacy were identified and it was considered retrospectively that the design could have been conducted as an adaptive enrichment design.

		The multiple-iteration bias-adjusted estimator tends to provide a less conservative estimation with respect to the single-iteration, and has similar variance and MSE with respect to the shrinkage estimators.<br><br>The first shrinkage estimator (S1) has a noticeable bias which also varies substantially from one sub-population to another, but the very low variance, resulting in the very low MSE.<br><br>The second shrinkage estimator (S2) performs similarly to S1, returning better performances in some cases and worse performances in other ones		
Optimised point estimators for multi-stage single-arm phase II oncology trials<br><br>Grayling and Mander (2022)<br><br>https://doi.org/10.1080/10543406.2022.2041656	Multi-stage single-arm design with dichotomous outcomes. Allows for early stopping for efficacy and futility<br><br>Compares the UMVUE with proposed estimators that minimise a weighted sum of the absolute bias and the RMSE over the parameter space (subject to additional constraints)<br><br>Focuses on unconditional bias and RMSE	The proposed estimators retained low bias across a wide range of response rates, specifically those that should be more realistic, and reduced the RMSE for certain response rates by a large amount compared to the UMVUE. Especially strong performance was seen in the two-stage setting.	No	Example based on phase II study of chemotherapy (https://doi.org/10.1007/s00280-016-2973-2)

3.2 Bias-reduced estimation

Paper title & authors	Trial context	Advantages / limitations	Code/software available?	Case studies?
On the Bias of Maximum Likelihood Estimation Following a Sequential Test<br><br>Whitehead (1986)<br><br>https://www.jstor.org/stable/2336521	The bias of maximum likelihood estimates calculated at the end of a sequential procedure is investigated<br><br>Proposes bias-adjusted MLE	"It would be unwise to use a conventional analysis on data collected from a sequential probability ratio test or a triangular test"<br><br>"When such designs result in a test of maximum sample size there may well be little bias involved in a conventional analysis. The methods of this paper could be used to quantify the bias, and to provide reassurance on this point."	No	None
Supplementary Analysis at the Conclusion of a Sequential Clinical Trial<br><br>Whitehead (1986)<br><br>https://www.jstor.org/stable/2531197	Explores two approaches (conditional and unconditional) to analyse secondary responses in a group sequential trial<br><br>Proposes bias-adjusted MLE	No comparisons made between estimators	No	Yes (three examples of sequential trials)
Stopping rules and estimation problems in clinical trials	Proposes a Bayesian method for assessing the plausible range of true	This approach is particularly useful for producing shrinkage of the unexpectedly large and imprecise observed treatment	No	Post-myocardial infarction trial

Hughes & Pocock (1988)<br><br>https://doi.org/10.1002/sim.4780071204	treatment effect for any trial based on interim results	effects that arise in clinical trials that stop early		
Improved approximation for estimation following closed sequential tests<br><br>Kim (1988)<br><br>https://doi.org/10.1093/biomet/75.1.121	Focuses on estimating the mean of a normal distribution with known variance following a class of sequential tests studied by Woodroofe<br><br>Proposes modified MLE, median unbiased estimator, and midpoint of a 90% confidence interval	The MLE and its modification are very sensitive to the magnitude of the stopped random walk, while the median unbiased estimator and the midpoint of the 90% confidence interval are not and are thus robust<br><br>However modified MLE performs best in terms of bias and MSE	No	None
Point estimation following group sequential tests<br><br>Kim (1989)<br><br>https://www.jstor.org/stable/2531502	Considers three point estimators for a normal mean following group sequential tests: the MLE, MUE and the midpoint of an exact 90% confidence interval	MUE and midpoint estimator have a “great reduction in bias”, but can have an increase in the MSE and variance. However, the reduction in bias is to a much greater degree than the increase in variance.<br><br>Midpoint estimator seems consistently better than MUE in terms of bias	No	None
The bias of the sample proportion following a group sequential phase II clinical trial	Investigates numerically the bias of the MLE of the binomial response probability, p, in group sequential phase II clinical trials and finds its	If one does not mind a bias of 0.025, one may find the sample proportion a suitable estimator for p because of its simplicity and easy explanation	No	None

Chang et al. (1989)<br><br>https://doi.org/10.1002/sim.4780080505	magnitude is less than 0.025 in all cases investigated<br>Applies Whitehead's idea to propose a bias-adjusted estimator that reduces the bias substantially and reduces the MSE as well in a certain range of p<br>Evaluates the UMVUE	If one is concerned with bias, the bias-adjusted estimator may be a good choice<br>The UMVUE has a higher MSE than the sample proportion or the bias-adjusted estimator for most values of *p*		
Practical problems in interim analyses, with particular regard to estimation<br><br>Pocock & Hughes (1989)<br><br>https://doi.org/10.1016/0197-2456(89)90059-7	Proposes a Bayesian "shrinkage" method of analysis to help quantify the extent to which surprisingly large point and interval estimates of treatment difference in clinical trials that stop early should be moderated		No	None
The bias of the sample proportion following a group sequential phase II clinical trial<br><br>Chang et al. (1989)<br><br>https://doi.org/10.1002/sim.4780080505	Investigates numerically the bias of the MLE of the binomial response probability, p, in group sequential phase II clinical trials and finds that its magnitude is less than 0.025 in all cases investigated<br>Applies Whitehead's idea to propose a bias-adjusted estimator that reduces the bias substantially and reduces the MSE as well in a certain range of p<br>Evaluates the UMVUE	If one does not mind a bias of 0.025, one may find the sample proportion a suitable estimator for p because of its simplicity and easy explanation<br>If one is concerned with bias, the bias-adjusted estimator may be a good choice	No	None

Estimation following sequential tests involving data-dependent treatment allocation<br><br>Coad (1994)<br><br>https://www.jstor.org/stable/24305541	Derives an expression for the bias (and variance) of the MLE based on Brownian motion approximations when a sequential test is used for two treatments. For normal responses, the approximation works well for several data-dependent allocations.<br><br>Mentions that Whitehead's idea can then be used to correct for bias, but no simulation results are given	In theory expressions should be good approximation for any data-dependent allocation rule used	No	None
Sequential estimation for two-stage and three-stage clinical trials<br><br>Coad (1994)<br><br>https://doi.org/10.1016/0378-3758(94)00033-6	Dervies expressions for the bias (and variance) of the MLE for the treatment difference for a two and three-stage trial with treatment selection (drop-the-loser design). Also considers the case with a linear time trend in the data.<br><br>Mentions that Whitehead's idea can then be used to correct for bias, but no simulation results are given		No	None
Point and interval estimation following a sequential clinical trial<br><br>Todd et al. (1996)<br><br>https://jstor.org/stable/2337615	Discusses two estimation techniques not based on orderings and modifies them to obtain improved accuracy<br>Provides a bias-adjusted MLE and median-unbiased estimator, together with a new and general method for setting confidence limits after a		No	None

	sequential clinical trial<br>Demonstrates accuracy of the methodology after a triangular test and an O'Brien & Fleming test through simulation			
Continuous and group sequential conditional probability ratio tests for phase II clinical trials<br><br>Tan & Xiong (1996)<br><br>https://doi.org/10.1002/(SICI)1097-0258(19961015)15:19%3C2037::AID-SIM339%3E3.0.CO;2-Z	Provides a bias-adjusted estimator of the success rate after group sequential stopping in single-arm clinical trials with binary endpoints		No	Three phase II studies in breast cancer
Estimating and reducing bias in group sequential designs with Gaussian independent increment structure<br><br>Pinheiro & DeMets (1997)<br><br>https://jstor.org/stable/2337655	Considers methods for estimating and reducing the bias of treatment difference estimators in group sequential designs with Gaussian independent increment structure<br>Derives an analytical expression for the bias and gives an easy-to-calculate approximate bound for its variation, and a simulation estimate of the bias, based		No	None

	on a Gaussian independent increment structure<br>Describes a related bias reduced estimator			
On the bias of estimation of a Brownian motion drift following group sequential tests<br><br>Li & DeMets (1999)<br><br>https://jstor.org/stable/24306627	Derives an analytical expression of the bias of the MLE for a group sequentially monitored Brownian motion process based on the alpha spending method of Lan and DeMets<br>Studies how the Brownian motion drift and various alpha spending functions and interim analysis patterns affect the bias<br>Describes a bias adjusted estimator and investigates its properties	Through this formula the bias can be evaluated exactly by numerical integration	Yes (FORTRAN code)	None
Conditional estimation following a group sequential clinical trial<br><br>Troendle and Yu (1999)<br><br>https://www.tandfonline.com/doi/abs/10.1080/03610929908832376	Gives three estimation methods to reduce the conditional bias in estimating the treatment difference for a group sequential trial with normally distributed outcomes, where the conditioning is on the stopping time	Shows through simulation that unconditionally unbiased estimators remain unbiased by overestimating the effect when there is early stopping, while underestimating the effect when the trial stops late<br><br>Proposed conditional estimators reduce the conditional bias, and can have similar conditional MSE to the usual MLE	No	None

Estimation following extension of a study on the basis of conditional power<br><br>Denne (2000)<br><br>https://doi.org/10.1081/BIP-100101018	Demonstrates that, in a two-stage design conditional power design procedure in which the target total sample size is dependent upon the data observed at the first stage, the MLE of the parameter of interest upon completion may be biased, and that this bias is similar in direction and magnitude to that commonly associated with estimation following a group sequential test with predetermined target total sample size<br>Shows how a bias adjusted estimate may be formed		No	National Heart, Lung, and Blood Institute's Type II Coronary Intervention Study
Flexible interim analyses in clinical trials using multistage adaptive test designs<br><br>Wassmer et al. (2001)<br><br>https://doi.org/10.1177/009286150103500410	Just a review but contains a section on point estimation and two examples		No	Superiority and non-inferiority trial
Conditional point estimation in adaptive group sequential test designs<br><br>Coburger & Wassmer (2001)	States that Whitehead's bias adjusted estimate for triangular designs is not feasible in adaptive designs although it is feasible in group sequential designs, and that it wastes information because it does not use the information at which stage the trial was stopped	The modified estimate achieves an improvement in group sequential designs and shows similar results in adaptive designs	No	None

https://doi.org/10.1002/1521-4036(200111)43:7%3C821::AID-BIMJ821%3E3.0.CO;2-F	Presents a modification which does use this information and is applicable to adaptive designs			
An improved method of evaluating drug effect in a multiple dose clinical trial<br><br>Shen (2001)<br><br>https://doi.org/10.1002/sim.842	Considers selecting the dose with the highest response rate in either a one or two-stage trial, using normal approximations<br><br>Proposes subtracting the conditional bias, where the estimate of the bias is given by a step function with arbitrarily-chosen step width gamma.	Focuses on construction of CIs and type I/II error rates<br><br>See also discussion in Stallard et al. (2008) for the case with two doses	No	None
On Sample Size and Inference for Two-Stage Adaptive Designs<br><br>Liu and Chi (2001)<br><br>https://doi.org/10.1111/j.0006-341X.2001.00172.x	Considers a general two-stage adaptive design, and proposes a median-unbiased estimator	No closed-form solution is given	No	None

Estimation following group-sequential response-adaptive clinical trials<br><br>Morgan (2003)<br><br>https://doi.org/10.1016/S0197-2456(03)00062-X	Considers one-sided group-sequential tests with response-adaptive sampling developed by Jennison and Turnbull is used to investigate which of the treatments has the larger mean response<br>Studies an approximation to the bias of the MLE of the treatment mean difference based on the work of Whitehead	Could use the approximation to the bias to construct bias-adjusted estimator	No	None
Sample size reassessment in adaptive clinical trials using a bias corrected estimate<br><br>Coburger & Wassmer (2003)<br><br>https://doi.org/10.1002/bimj.200390051	Presents a bias adjusted estimator which allows a more exact sample size determination based on the conditional power principle than the naive sample mean does	Numerical problems can occur when calculating adjusted estimators, and observations close to the critical boundaries can lead to unreasonably extreme adjusted estimators	No	None
Practical midcourse sample size modification in clinical trials<br><br>Proschan et al. (2003)<br><br>https://doi.org/10.1016/S0197-2456(02)00240-4	Considers a two-stage adaptive design with sample size re-estimation where the second stage sample size is based on the first-stage results<br><br>Proposes median-unbiased estimators based on the midpoint of confidence intervals	No results given	No	Example based on a Phase I trial in hypertension prevention (https://doi.org/10.1001/jama.1992.03480090061028)

Conditional bias of point estimates following a group sequential test<br><br>Fan et al. (2004)<br><br>https://doi.org/10.1081/BIP-120037195	Investigates the conditional and marginal biases with focus on the conditional one upon the stopping time in estimating the Brownian motion drift parameter in sequential trials<br>Finds that the conditional bias may be very serious for existing point estimation methods, even if the unconditional bias is satisfactory<br>Proposes new conditional estimators which can significantly reduce the conditional bias from unconditional estimators	The proposed estimators can provide a much smaller conditional bias and MSE than the naive MLE and Whitehead's bias reduced estimator	No	Two cardiovascular trials (MERIT-HF and COPERNICUS)
Conditional maximum likelihood estimation following a group sequential test<br><br>Liu et al. (2004)<br><br>https://doi.org/10.1002/bimj.200410076	Considers estimation after a group sequential test, where an estimator that is unbiased or has small bias may have substantial conditional bias<br>Derives the conditional MLEs of both the primary parameter and a secondary parameter, and investigate their properties within a conditional inference framework	The method applies to both the usual and adaptive group sequential test designs	No	Amilial adenomatous polyposis trial

Estimation of a parameter and its exact confidence interval following sequential sample size reestimation trials<br><br>Cheng and Shen (2004)<br><br>https://onlinelibrary.wiley.com/doi/full/10.1111/j.0006-341X.2004.00246.x	Derives a bias-adjusted estimator (based on the method of moments) for the mean of a normally distributed outcome following the self-designing group sequential trial by Shen and Fisher (1999, Biometrics 55, 190-197)	Extensive simulation studies show that the naive estimator considerably overestimates the true parameter, whereas the proposed point estimates are nearly unbiased with practical sample sizes.<br><br>The variances of the proposed estimators are similar to the naive estimator.<br><br>The proposed estimates are shown to be consistent.<br>The computation of the estimates is "straightforward".	No	None
On the estimation of the binomial probability in multistage clinical trials<br><br>Jung and Kim (2004)<br><br>https://doi.org/10.1002/sim.1653	Multistage sequential design with binary responses<br><br>Derives analytical formula for the UMVUE<br><br>Considers bias-adjusted MLE	In a two-stage setting, the MLE has smaller MSE for smaller p-values than UMVUE, but larger MSE for larger p-values. There appears to be some efficiency loss with the UMVUE as compared to the MLE, particularly for optimal designs, a reasonable price for unbiasedness.<br><br>Bias-adjusted MLE can have larger MSE than the UMVUE in some situations.	No	None

Supplementary analysis of probabilities at the termination of a group sequential phase II trial<br><br>Liu et al. (2005)<br><br>https://doi.org/10.1002/sim.1990	Considers estimation of various probabilities after termination of a group sequential phase II trial<br>Shows that the conventional MLE (sample proportion) is biased<br>Proposes two alternative estimators to correct for bias, a bias-reduced estimator obtained by using Whitehead's bias-adjusted approach, and an unbiased estimator from the Rao-Blackwell method of conditioning	All three estimation procedures are shown to have certain invariance properties in bias<br>Estimators of a probability and their bias and precision can be evaluated through the observed response rate and the stage at which the trial stops, thus avoiding extensive computation	No	None
A simple and efficient bias-reduced estimator of response probability following a group sequential phase II trial<br><br>Guo & Liu (2005)<br><br>https://doi.org/10.1081/BIP-200067771	Proposes an estimator of response rate in a phase II trial with interim futility analyses by subtracting the estimated bias directly from the sample proportion	The proposed estimator is simple, intuitive, and easy to compute	No	None
Point estimates and confidence regions for sequential trials involving selection	Group sequential trial where several treatments are compared with a control in the first stage, and the most promising treatment is carried forward to the subsequent stages	The bias-adjusted estimator has a considerably smaller bias than the MLE, which tends to be an overestimate	No	Case study based on phase III clinical trial for Alzheimer's disease

Stallard and Todd (2005)<br><br>https://doi.org/10.1016/j.jspi.2004.05.006	Proposes a bias-adjusted estimator (following the approach of Whitehead)			
p-Value Calculation for Multistage Phase II Cancer Clinical Trials<br><br>Jung et al. (2006)<br><br>https://doi.org/10.1080/10543400600825645	Investigates some approaches to p-value calculation in analyzing multi-stage Phase II clinical trials that have a binary variable	Whatever estimator is used, the p-values depend on the ordering, but not on the estimates. So, two estimators will result in exactly the same p-value if they have the same ordering. Chang et al. (1989) correct the bias of the MLE using Whitehead's (1986) approach. The bias-corrected estimator has exactly the same ordering as the MLE, so the two estimators result in the same p-value.<br><br>Further, both MLE and UMVUE increase in s for each m. Hence, the two estimators will have the same ordering as long as their ordering matches at the boundaries. Otherwise can have discordant p-values.<br><br>Only the UMVUE ordering provides p-values with desirable properties.	No	None
Adaptive design: Estimation and inference with censored data in a semiparametric model	Proposes a bias-adjusted parameter estimator for the treatment effect and its asymptotic CI at the end of the trial in the adaptive design for censored survival data with or without adjusting for risk	The computation of the estimates is straightforward<br>The asymptotic CIs have reasonable nominal probability of coverage, and the proposed point estimators are nearly	No	Colon cancer trial

Shen and Cheng (2007)<br><br>https://doi.org/10.1093/biostatistics/kxl011	factors	unbiased with practical sample sizes		
On Bayesian estimators in multistage binomial designs<br><br>Bunouf and Lecoutre (2008)<br><br>https://doi.org/10.1016/j.jspi.2008.02.014	Considers a new class of Bayesian estimators for a proportion in multistage binomial designs<br><br>Compares new Bayesian estimators to Whitehead's and UMVUE	Exact calculations of posterior mode/mean can be computationally intensive<br><br>For two-stage designs, easy-to-use approximation of the posterior mode is given<br><br>Compared to MLE, the advantage of the Bayesian estimators and Whitehead estimator is substantial. In compensation for its unbiasedness, UMVUE exhibits less advantageous characteristics in terms of relative efficiency<br><br>In setting of planning experiments to assess the probability of rare events, Bayesian estimators have small bias and strongly increasing relative efficient compared to MLE	No	None
Estimation following selection of the largest of two normal means	Compares the estimators of Shen (2001) and Stallard & Todd (2005) when there are two treatment (doses)	Shows that the Stallard & Todd estimator has infinite conditional expectation.<br><br>Shen's estimator does not uniformly	No	None

Stallard et al. (2008)<br><br>https://doi.org/10.1016/j.jspi.2007.05.045	Proposes a family of approximately conditionally unbiased estimates	improve on bias or MSE<br><br>Proposed family of estimator has smaller bias than Shen's estimator for range of true parameter values and can sometimes have smallerMSE		
Point and Interval Estimation of Primary and Secondary Parameters in a Two-Stage Adaptive Clinical Trial<br><br>Liu et al. (2008)<br><br>https://doi.org/10.1080/10543400701697125	Considers a two-stage adaptive trial with normally-distributed observations. At the interim analysis a new sample size is calculated based on the primary endpoint of interest.<br><br>For the primary endpoint, compares the MLE, Whitehead's bias-adjusted approach, an unbiased Rao-blackwellized estimator, and two weighted estimators<br><br>For the secondary endpoint, compares a bias-adjusted estimate and an unbiased Rao-blackwellized estimator	For the primary endpoint, the bias-adjusted estimate always has smaller bias than the other estimators (apart from the unbiased estimator) whereas the maximum likelihood estimate has the largest<br><br>The unbiased estimator tends to have larger MSE, whereas the others have comparable MSE.<br><br>Overall, the bias-adjusted estimator tends to be the best among the estimators considered	No	None
Conditional estimation of sensitivity and specificity from a phase 2 biomarker study allowing Early termination for futility	Two-stage design testing the sensitivity of dichotomous biomarker, with early stopping for futility<br><br>Derives and compares the UMVUE, UMVCUE, median-adjusted estimator and mean-adjusted estimator	Naive estimator can have "substantial" upward bias<br><br>UMVCUE is unbiased and, when early stopping is unlikely, its precision is comparable with the naive estimator	No	None

Pepe et al. (2009)<br><br>https://doi.org/10.1002/sim.3506		Mean- and median-adjusted estimators have similar performance to UMVCUE, but their computation is more difficult<br><br>UMVUE can be conditionally biased		
Parameter estimation following an adaptive treatment selection trial design<br><br>Luo et al. (2010)<br><br>https://doi.org/10.1002/bimj.200900134	Two-stage drop-the-losers trial, where number of responses follow a Binomial distribution<br><br>Derives bias-adjusted MLE based on method of moments, and compares this with naive estimator and Rao-Blackwell estimator	Bias-adjusted MLE has to be calculated numerically<br><br>Simulations suggest that the bias-adjusted MLE has relatively low RMSE and acceptably small bias – generally smaller than that of the naive estimator and practically comparable with the Rao–Blackwell estimator. Its MSE performed a bit better than that of the Rao–Blackwell-type estimator. In addition, it can be applied for the one-stage situation	No	Hypothetical example based on study in colorectal cancer
Statistical Inference after an Adaptive Group Sequential Design: A Case Study<br><br>Tremmel (2010)<br><br>https://doi.org/10.1177%2F009286151004400506	Case study on inference after the 02CLLIII trial in chronic leukemia, which was an adaptive group sequential design<br><br>Uses bias-adjusted estimator as proposed by Coburger & Wassmer (2001)	“The flexibility offered by the adaptive features renders statistical inference more difficult and less precise”<br><br>Conditional bias is much more severe than the unconditional bias, but the direction of bias is conservative (underestimates)	No	02CLLIII trial in chronic leukemia, which was an adaptive group sequential design

		If rigid statistical decision rules were not followed, bias cannot be studied by simulation -> use doubly conditioning approach by Coburger & Wassmer (2001), conditioning on all of past history		
Likelihood inference for a two-stage design with treatment selection<br><br>Bebu et al. (2010)<br><br>https://doi.org/10.1002/bimj.200900170	A conditional likelihood-based approach is proposed for the parameters of interest in a two-stage design with treatment selection after the first stage and normally distributed responses.<br><br>Compares conditional MLE (cMLE) with naive MLE and UMVCUE	The bias of the cMLE estimator is also reasonably small, but always negative. On the other hand, the naive MLE has a larger bias that is always positive<br><br>However naive MLE has smallest MSE	No	None
An MSE-reduced estimator for the response proportion in a two-stage clinical trial<br><br>Li (2011)<br><br>https://doi.org/10.1002/pst.414	Considers estimation for a two-stage single arm trial with binary responses, with early stopping for futility only.<br><br>Compares the MLE with a proposed MSE weighted estimator	Compared with the MLE, the proposed estimator can reduce the bias substantially and improve the MSE.	No	None
Conditional estimation after a two-stage diagnostic biomarker study that allows	Discuss conditional estimation of parameters of interest after a two-stage	Mean and median adjusted estimators have similar standard errors to UMVCUE	No	None

early termination for futility<br><br>Koopmeiners et al. (2012)<br><br>https://doi.org/10.1002/sim.4430	study that allows early termination for futility<br><br>Compares conditional mean adjusted MLE, conditional median adjusted MLE and the UMVCUE	MLE has the smallest standard error when true parameter is small and there is a high probability of termination, but there is little difference when true parameter is larger and early termination is rare		
What inference for two-stage phase II trials?<br><br>Porcher & Desseaux (2012)<br><br>https://bmcmedresmethodol.biomedcentral.com/articles/10.1186/1471-2288-12-117	Compares different approaches for point and confidence intervals estimation for Simon's two-stage design	For point estimation, the UMVUE was unbiased both when the actual number of patients recruited was equal to or differed from the pre-planned value. The bias corrected estimator had negligible bias and slightly lower RMSE than the UMVUE only when the true response rate was close to its value under the null hypothesis. "Both estimators performed better than the others and can thus be recommended"	No	None
Estimation of Treatment Effect Following a Clinical Trial with Adaptive Design<br><br>Luo et al. (2012)<br><br>https://doi.org/10.1080/10543406.2012.676534	Generic two-stage adaptive trial comparing multiple treatments against a control<br><br>Models clinical trial data as a marked pointed process (i.e. a stochastic process framework)<br><br>Proposes bias-adjusted estimator using a general estimating equation to match the first conditional moment	Proposed estimator can be used for any probability distribution<br><br>Proposed estimator has smaller bias than the naive estimator in many scenarios, but has a higher MSE and can have a substantial negative bias	No	None

Shrinkage estimation in two-stage adaptive designs with midtrial treatment selection<br><br>Carreras & Brannath (2013)<br><br>https://doi.org/10.1002/sim.5463	Proves that selection bias of the MLE is maximal when all treatment effects are equal and the most-promising treatment is selected<br><br>Extends previous work on Lindley's estimator for single-stage multi-armed trials with four or more treatments and post-trial treatment selection	A simple two-stage version of Lindley's estimator has uniformly smaller Bayes risk than the MLE when assuming an empirical Bayesian framework with independent normal priors for the group means<br><br>The shrinkage estimators perform well compared with the MLE and previously suggested bias-adjusted estimators in terms of selection bias and MSE	No	None
Conditionally unbiased estimation in phase II/III clinical trials with early stopping for futility<br><br>Kimani et al. (2013)<br><br>https://doi.org/10.1002/sim.5757	Seamless phase II/III clinical trials with normally distributed endpoint<br><br>Derives unbiased estimator based on Rao-Blackwellisation, and a bias-adjusted estimator	The bias-adjusted estimator overcorrects for bias, and the overcorrection increases with selection time but decreases as the value of the futility boundary increases. The naive estimator has the lowest MSE at all selection times for all scenarios. The unbiased estimator and the bias-adjusted estimator have approximately equal MSE	No	Case study based on a comparison of three doses of an experimental drug for generalized anxiety disorder with a placebo
Empirical Bayes estimation of the selected treatment mean for two-stage drop-the-loser trials: a meta-analytic approach	Two-stage drop-the-loser trial with normally distributed effect estimates<br><br>Proposes the use of various forms of shrinkage estimation (which can be viewed as Empirical Bayes estimators)	Shrinkage estimators are shown to perform favourably compared with MLE (and the shrinkage estimator proposed by Carreras and Brannath) in terms of bias and MSE<br><br>However, they necessitate either explicit estimation of an additional parameter	No	None

Bowden et al. (2013)<br><br>https://doi.org/10.1002/sim.5920		measuring the heterogeneity between treatment effects or a quite unnatural prior distribution for the treatment effects that can only be specified after the first stage data has been observed		
Conditionally unbiased and near unbiased estimation of the selected treatment mean for multistage drop-the-losers trials<br><br>Bowden and Glimm (2014)<br><br>https://dx.doi.org/10.1002%2Fbimj.201200245	Derive unbiased and near-unbiased estimates for the multistage drop-the-losers design<br><br>Unbiased estimator based on Rao-Blackwellisation, and near-unbiased estimator based on conditional MLE	Unbiased estimator has a large MSE<br><br>Conditional MLE computationally intensive. Overcorrects for bias but has reduced MSE compared with unbiased estimator	Yes (R code)	None
An evaluation of inferential procedures for adaptive clinical trial designs with pre-specified rules for modifying the sample size<br><br>Levin et al. (2014)<br><br>https://doi.org/10.1111/biom.12168	Extends group sequential orderings of the outcome space based on the stage at stopping, likelihood ratio statistic, and sample mean to the adaptive setting in order to compute median-unbiased point estimates and exact CIs	The bias adjusted mean demonstrates the lowest MSE among candidate point estimates<br><br>A conditional error-based approach in the literature has the benefit of being the only method that accommodates unplanned adaptations	Yes (R code)	None

On random sample size, ignorability, ancillarity, completeness, separability, and degeneracy: Sequential trials, random sample sizes, and missing data<br><br>Molenberghs et al. (2014)<br><br>https://doi.org/10.1177/0962280212445801	Considers generic trial settings where the sample size is a random variable depending on the data being collected (e.g. missing data, sequential trials, informative cluster size)<br><br>Compares the sample average vs the conditional likelihood estimator	In the cases of univariate incomplete data and sequential trials, the sample average is biased in small samples but asymptotically unbiased, both conditionally and marginally. The maximum conditional likelihood estimator is unbiased but the ordinary sample average still has the smaller mean squared error<br><br>“For sequential trials, there has been long-standing confusion and controversy regarding the (in)appropriateness of the sample average when estimating a parameter after such a trial. Our results show that the ordinary sample average, while small-sample biased and not uniform minimum variance unbiased, is perfectly acceptable. This should be seen against the background of the conditional likelihood estimator. Even though the latter is small-sample unbiased, it suffers from a slightly increased mean square error. Thus, in conclusion, while some familiar properties no longer hold, estimation after sequential trials is more straightforward than commonly considered and there is less need for complicated, modified estimators than perhaps generally thought, given that the ordinary sample average can be used without trouble.”	No	None

Estimation after a group sequential trial<br><br>Milanzi et al. (2015)<br><br>https://doi.org/10.1007/s12561-014-9112-6	Shows that the sample average is a justifiable estimator, in the sense that it follows from joint likelihood estimation, and it is consistent and asymptotically unbiased<br>Shows why simulations can give the false impression of bias in the sample average when considered conditional upon the sample size	Argues that no corrections need to be made to estimators following sequential trials<br>When small-sample bias is of concern, the conditional likelihood estimator (CLE) provides a relatively straightforward modification to the sample average<br>Classical likelihood-based SEs and CIs can be applied, obviating the need for technical corrections	No	None
Properties of estimators in exponential family settings with observation-based stopping rules<br><br>Milanzi et al. (2016)<br><br>https://dx.doi.org/10.4172%2F2155-6180.1000272	Considers general trial settings with stochastic stopping rules (including group sequential designs) where the outcome follows a one-parameter exponential family	An unbiased estimator follows from the conditional likelihood, where the conditioning is on the (non-ancillary) sample size.<br>The conditional estimator has larger mean squared error than the ordinary sample average for sufficiently large sample size<br><br>"The sample average is a valid and sensible estimator, contrary to some claims in the sequential-trial literature, for stochastic and deterministic stopping rules"	No	None
Quantifying over-estimation in early stopped clinical trials and the "freezing effect" on subsequent research	Studies the effect (in terms of bias) of early stopping via simulations, considering two-arm randomized clinical	Across the trials whose true effects are sampled from a uniform distribution, estimates from trials that stop early for	No	None

Wang et al. (2016)<br><br>https://doi.org/10.1177%2F1740774516649595	trials with a binary primary outcome and group sequential stopping rules<br><br>Also considers using Bayesian estimates of treatment effects under different priors	efficacy deviate minimally from the simulation truth (median bias of 0.005).<br><br>Over-estimation becomes appreciable only when the true effect is close to the null value 0 (median bias is 0.04) or when stopping happens with 40% information or less; however, stopping under these situations is rare.<br><br>There is a slight reverse bias of the estimated treatment effect (median bias is -0.002) among trials that do not cross the early stopping boundaries but continue to the final analysis.<br><br>In contrast, Bayesian estimation of the treatment effect shrinks the estimate from trials stopping early and pulls back under-estimation from completed trials, largely rectifying any over-estimation among trials that terminate early		
Point estimation and p-values in phase II adaptive two-stage designs with a binary endpoint<br><br>Kunzmann and Kieser (2017)	Adaptive two-stage single-arm trial with a binary endpoint. The stage 2 sample size is determined based on the results of stage 1.<br><br>Derives a Rao-Blackwellised estimator	None of the point estimators previously known from the literature guarantees compatibility with the test decision	No	None

https://doi.org/10.1002/sim.7200	Proposes an optimal compatible (OC) estimator, which minimises the MSE subject to compatibility with the decision rule and monotonicity conditions	OC reduces MSE of MLE except for very small or very large values of the success probability<br><br>Rao-Blackwellised estimator is unbiased but has the highest MSE<br><br>OC reduces absolute bias compared with MLE except for small values of the success probability, where is has a substantial positive bias		
Group sequential control of overall toxicity incidents in clinical trials - Non-Bayesian and Bayesian approaches<br><br>Yu et al. (2016)<br><br>https://doi.org/10.1177%2F0962280212440535	Proposes group sequential toxicity monitoring strategies to control overall toxicity incidents below a certain level<br><br>Uses Whitehead's bias-adjusted approach to estimate the sample proportion	The bias of the sample proportion tends to be substantial for the toxicity control designs proposed in this article	Programs (in Mathematica) available from the authors upon request	None
Comparison of conditional bias-adjusted estimators for interim analysis in clinical trials with survival data<br><br>Shimura et al. (2017)<br><br>https://doi.org/10.1002/sim.725	Reviews the characteristics of the conditional mean-adjusted estimator (CMAE), conditional median-unbiased estimator, conditional uniformly minimum variance unbiased estimator (CUMVUE), and weighted estimator and their CIs and compares their conditional bias, overall bias, and conditional MSE in clinical trials with survival endpoints	The CMAE reduces conditional bias and shows relatively small conditional MSE in trials terminated at the interim analysis<br><br>The CUMVUE has less bias and an acceptable conditional coverage probability in trials not terminated early	No	Breast cancer trial (CLEOPATRA)

8				
Conditional estimation in two-stage adaptive designs<br><br>Broberg & Miller (2017)<br><br>https://doi.org/10.1111/biom.12642	Considers a design which permits raising the sample size when interim results look rather promising, and which retains the originally planned sample size when results look very promising<br>Compares the unconditional MLE, the conditionally unbiased Rao-Blackwell estimator (UMVCUE), the conditional median-unbiased estimator, and the conditional MLE with and without bias correction	The MLE performs well in the simulations in terms of MSE. However, it MLE does not possess any optimality features in the conditional inference setting.<br>The difference between the four conditional estimators was quite small in the scenarios considered. This was also reflected in terms of their bias (they had little or no bias) and their variance, which was similar.<br>The conditional Rao–Blackwell estimator has the advantage that it is unbiased in construction and has an explicit representation making computation simpler.	No	Schizophrenia trial
Estimation in multi-arm two-stage trials with treatment selection and time-to-event endpoint<br><br>Brückner et al. (2017)<br><br>https://doi.org/10.1002/sim.7367	Adapts several methods for reducing the selection bias that have been proposed for normal endpoints to time-to-event data, including an iterative method based on the estimated conditional selection biases and a shrinkage approach based on empirical Bayes theory	The maximum partial-likelihood estimator of the log hazard ratio of the selected treatment overestimates the true treatment effect<br>All bias reduction methods tend to overcorrect the bias, and only the shrinkage methods can reduce the MSE	No	FOCUS trial

Point estimation in adaptive enrichment designs<br><br>Kunzmann et al. (2017)<br><br>https://doi.org/10.1002/sim.7412	Two-stage adaptive enrichment design where a binary biomarker is applied to select the patient population investigated in stage 2. The outcomes are assumed to be normally distributed with known variance.<br><br>Proposes alternatives to the MLE:<br><br>1. Empirical Bayes Estimator<br>2. Parametric Bootstrap Estimator<br>3. Conditional Moment Estimator<br>4. Hybrid estimator (UMVCUE and MLE or CME)	EBE and PBE exhibit a higher bias than the MLE in many situations<br><br>CME and hybrid consistently reduce the bias but there is a price to be paid in terms of MSE	No	Illustrative example based on MILLY phase II study in asthma
Conditional estimation using prior information in 2-stage group sequential designs assuming asymptotic normality when the trial terminated early<br><br>Shimura et al. (2018)<br><br>https://doi.org/10.1002/pst.1859	Proposes a new estimator for adjusting the conditional bias of the treatment effect for trials that are terminated early for efficacy or futility by extending the idea of the conditional mean-adjusted estimator (CMAE)<br><br>The estimator is calculated by weighting the maximum likelihood estimate obtained at the interim analysis and the effect size prespecified when calculating the sample size	The conditional bias of the proposed estimator is smaller than that of the CMAE when the information time at the interim analysis is small<br><br>The MSE of the proposed estimator is also smaller than that of the CMAE	No	Phase II chemotherapy study
Underestimation of treatment effects in sequentially monitored clinical trials that did not stop early for benefit	Group sequential trial comparing two treatment groups	Unconditional MLE has very little bias when there is little or no treatment effect, but increases to quite substantial bias when the treatment effect is more substantial. The bias associated with larger treatment	Yes (R code)	GUSTO study for heart attack. Cost effectiveness was important

Marschner & Schou (2019)<br><br>https://doi.org/10.1177/0962280218795320	Calculates analytical expression for conditional bias of MLE in trials that do not stop early<br><br>Proposes conditional MLE	effects corresponds to underestimation in excess of 20% when there is a single interim analysis, and in excess of 40% when there are three interim analyses.<br><br>Conditional MLE is very effective at rectifying the underestimation bias present in the unconditional MLE<br><br>Neither has uniformly smaller MSE		consideration -> unbiased estimate is crucial
Point estimation following two-stage adaptive threshold enrichment clinical trials<br><br>Kimani et al. (2018)<br><br>https://doi.org/10.1002/sim.7831	Develops point estimators for clinical trials that use the two-stage adaptive enrichment threshold design. The design consists of two stages, where in stage 1, patients are recruited in the full population. Stage 1 outcome data are then used to perform interim analysis to decide whether the trial continues to stage 2 with the full population or a subpopulation. The subpopulation is defined based on one of the candidate threshold values of a predictive biomarker.<br><br>Derives unbiased estimators (including UMVCUE), shrinkage estimators and bias-adjusted estimators	Depending on the scenario, the bias of the naive estimator of the treatment effect in the selected subpopulation is substantial and can be negative or positive.<br><br>Recommends use of the UMVCUE - although it has larger MSE than some estimators, the bias eradicated in most cases was larger than the difference in RMSEs<br><br>Since none of the estimators dominated in all simulation scenarios based on both bias and MSE, an alternative strategy would be to use a hybrid estimator where the estimator used depends on the subpopulation selected. This would require a simulation study of plausible scenarios before the trial.	No	None

Using Bayesian modeling in frequentist adaptive enrichment designs<br><br>Simon and Simon (2018)<br><br>https://doi.org/10.1093/biostatistics/kxw054	Proposes a framework for group sequential adaptive enrichment trials<br><br>Includes a frequentist hypothesis test at the end of the trial. However, it uses Bayesian methods to estimate the effect size.	Because an uninformative (and sometimes more mis-specified) prior is used, the effect size estimates are biased. In scenarios with more signal this bias is slight, however this bias can become significant.	No	Redesign of cetuximab trial
Assessment of Hazard Ratios in Oncology Clinical Trials Terminated Early for Superiority: A Systematic Review<br><br>Shimura et al. (2020)<br><br>https://jamanetwork.com/journals/jamanetworkopen/fullarticle/2767516	A systematic review of oncology clinical trials using group sequential designs with a single preplanned interim analysis as well as 2-arm randomized clinical trials that were subsequently stopped for efficacy reasons<br><br>198 abstracts were screened for eligibility, of which, 19 eligible clinical trials were identified as applicable to the bias-adjusted estimators<br><br>Compares conditional mean-adjusted estimator (CMAE) and weighted CMAE (WCMAE) with unadjusted hazard ratio (i.e. the MLE)	No trials actually used any bias-adjusted estimators in practice<br><br>The bias-adjusted estimators in large trials were similar to the unadjusted HR. However, the bias-adjusted estimators in the small trials were highly disparate from the unadjusted HRs.<br><br>For treatments with extremely positive effects, risks of overestimating unadjusted HR would be minimal. Conversely, larger differences between the unadjusted and bias-adjusted HRs were generally observed when the estimated HR was larger than 0.5<br><br>Authors recommend presenting bias-adjusted estimators with the MLE to data monitoring committees, especially when the trial is terminated early<br><br>Most standard statistical software cannot directly produce bias-adjusted estimators	No	Yes (19 clinical trials)

Estimation of treatment effect in 2-in-1 adaptive design and some of its extensions<br><br>Li et al. (2021)<br><br>https://doi.org/10.1002/sim.8917	Considers the 2-in-1 adaptive design which allows seamless expansion of an ongoing phase II trial into a phase III trial. More specifically, the role of the interim analysis is to make a decision whether to keep the study as a phase II trial or expand to a phase III trial. The endpoints for the interim and final analyses can be different.<br><br>Also considers two biomarker designs with a similar data structure: 1) expansion of a biomarker positive study to an all-comer study; 2) expansion of biomarker positive population in an all-comer study<br><br>Proposes and compares the naive, mean unbiased, median unbiased and conditionally bias-adjusted estimators in terms of overall (unconditional) bias and MSE as well as the conditional bias	The conditionally bias-adjusted estimator has smaller overall bias than the naive estimator<br>The mean unbiased estimator has larger RMSE then all other estimators, while the RMSE of the median unbiased and conditionally bias-adjusted estimator is comparable to that of the naive estimator<br><br>All estimators are conditionally biased, but conditionally bias-adjusted estimator has substantially smaller conditional bias.	No	None
A General Framework for the Analysis of Adaptive Experiments<br><br>Marschner (2021)<br><br>https://doi.org/10.1214/20-STS803	Presents a unifying formulation of adaptive designs and a general approach to their analysis, which is based on the partitioning of the overall unconditional information into its two component sources. More precisely, the unconditional likelihood can be expressed as the product of the design likelihood and the conditional likelihood	“Rather than advocating for or against unconditional inference over conditional inference in general, we will take the approach of presenting a framework for exploring the extent to which conditional bias is likely to be present within a given sample.”<br><br>Given the potential for conditional bias in the unconditional MLE, the conditional MLE	No	None

	(conditioned on the realised design)<br><br>Shows how to calculate the conditional MLE in general.<br><br>Proposes a penalised MLE, which weights the design likelihood. The penalised MLE can hence vary between the two extremes of the unconditional and conditional MLEs<br><br>Gives an example of the conditional and penalised MLE in the context of a RAR design	may be useful as a conditionally unbiased analysis tool.<br><br>In the RAR example, the conditional MLE can be very effective at eliminating the conditional bias that is present in the unconditional analysis. However, this comes at the cost of a loss of efficiency except for more extreme designs where the unconditional MLE can have a higher MSE. Meanwhile, the penalised MLE exhibits very little conditional bias and is not subject to substantial efficiency loss (compared with the unconditional MLE) when the realised design is close to its average.		
Optimised point estimators for multi-stage single-arm phase II oncology trials<br><br>Grayling and Mander (2022)<br><br>https://doi.org/10.1080/10543406.2022.2041656	Multi-stage single-arm design with dichotomous outcomes. Allows for early stopping for efficacy and futility<br><br>Compares the UMVUE with proposed estimators that minimise a weighted sum of the absolute bias and the RMSE over the parameter space (subject to additional constraints)<br><br>Focus on unconditional bias and RMSE	The proposed estimators retained low bias across a wide range of response rates, specifically those that should be more realistic, and reduced the RMSE for certain response rates by a large amount compared to the UMVUE. Especially strong performance was seen in the two-stage setting.	No	Example based on phase II study of chemotherapy (https://doi.org/10.1007/s00280-016-2973-2)
A simulation-based comparison of estimation methods for adaptive and classical group sequential	Considers estimation for adaptive group sequential designs. At the interim look, the trial is redesigned by adapting the sample size, number of future looks and the alpha-spending function, based on	Across all simulations, the MUE provides an essentially unbiased estimate.<br><br>For the two-stage trial example the naive estimate and RCI midpoint were unbiased,	Yes (R code)	Example of a planned clinical trial evaluating deep brian stimulation versus conventional therapy for the treatment

clinical trials<br><br>Nelson et al. (2022)<br><br>https://doi.org/10.1002/pst.2188	conditional power considerations.<br><br>Compares the naive estimate, the MUE (based on the backwards confidence interval) and the midpoint of the repeated confidence interval (RCI).	but for more than two stages these estimators were biased.		of Parkinson's disease (see https://doi.org/10.1111/j.0006-341X.2001.00886.x for details)
A comparison of estimation methods adjusting for selection bias in adaptive enrichment designs with time-to-event endpoints<br><br>Stefano et al. (2022)<br><br>https://doi.org/10.1002/sim.9327	Compares side-by-side the performances of six estimators of the treatment effects for two-stage enrichment ADs with time-to-event data:<br><br>● Naive estimator (MLE)<br>● UMVCUE<br>● Shrinkage estimators<br>● Bias-adjusted estimators<br><br>Focus is on conditional estimation (conditional on the selection made for the subgroups)	The naive estimator has the highest bias but very low variance, resulting in a moderate MSE compared to the other estimators.<br><br>The UMVCUEhas a small constant positive bias (which is not zero because of the correlation bias, but is reduced increasing the number of sub-populations) and the highest variance, resulting in the highest MSE.<br><br>The single-iteration bias-adjusted estimator has small bias, but more variance with respect to naive estimator,<br>resulting in comparable MSE with respect to the naive estimator.<br><br>The multiple-iteration bias-adjusted estimator tends to provide a less conservative estimation with respect to the single-iteration, and has similar variance and MSE with respect to the SI.	Yes (R code)	Case study based on a real trial in heart failure. The initial study was a group sequential design (without population selection). After the analysis, some subgroups with different efficacy were identified and it was considered retrospectively that the design could have been conducted as an adaptive enrichment design.

		The first shrinkage estimator (S1) has a noticeable bias which also varies substantially from one sub-population to another, but the very low variance, resulting in the very low MSE.<br><br>The second shrinkage estimator (S2) performs similarly to S1, returning better performances in some cases and worse performances in other ones		

3.3 Resampling-based approaches

Paper title & authors	Trial context	Advantages / limitations	Code/software available?	Case studies?
Bias reduction via resampling for estimation following sequential tests<br><br>Wang and Leung (1997)<br><br>https://doi.org/10.1080/07474949708836386	Focuses on estimating parameters following a sequential test.<br><br>The proposed resampling approach approximates the bias of the estimate using a bootstrap method. It requires bootstrapping the sequential testing process by resampling observations from a distribution based on the MLE. Each bootstrap process will give a new MLE, and the corresponding bootstrap mean can be used to calibrate the estimate.	An advantage of the new method over the existing methods is that the same procedure can be used under different stopping rules and different study designs.<br><br>Simulation results suggest that this method performs competitively with existing methods: “it is able to correct for almost all the biases induced by optional stopping and did so without any compromise in variance”	No	None
Bias reduction using stochastic approximation<br><br>Leung and Wang (1998)<br><br>https://doi.org/10.1111/1467-842X.00005	Studies stochastic approximation as a technique for bias reduction.<br><br>Gives an example in the context of sequential clinical trials	The proposed method does not require approximating the bias explicitly, nor does it rely on having iid data.<br><br>The performance of this method becomes less dependent on the behaviour of the original parameter estimate.<br><br>In example for sequential trials there was smaller residual bias than for bias-adjusted estimator, as well as a smaller variance than the MLE	No	None

		Convergence of the method is quick/computationally inexpensive		
Using the bootstrap for estimation in group sequential designs: An application to a clinical trial for nasopharyngeal cancer<br><br>Leblanc and Crowley (1999)<br><br>https://doi.org/10.1002/(SICI)1097-0258(19991015)18:19%3C2635::AID-SIM200%3E3.0.CO;2-7	Investigates a resampling method for bias correction in group sequential designs with censored survival data using logrank testing.<br><br>The method draws nested bootstrap samples of different sizes from the observed data in order to mimic the large sample independent increment property of statistics resulting from sequential designs	Relatively complicated and computationally intensive bootstrap procedure<br><br>Bootstrap procedure generally estimates the bias well, although can have some underestimation<br><br>“As noted by one referee, some researchers have philosophical reasons for not being interested in bias correction in clinical trials. For instance, since the likelihood does not depend on the stopping rule, some believe that bias correction based on frequentist notions is not of interest. Other reasons some may argue against bias correction could be based on the potentially much larger biases that exist if one is attempting to make statements about the larger population that would ultimately be treated. For instance, there may be differences in adherence, type of patient monitoring and, of course, patient population.”	No	Clinical trial for nasopharyngeal cancer
Group sequential enrichment design incorporating subgroup selection	Proposes an adaptive enrichment group sequential procedure. The design eliminates populations at the first stage that appear likely to derive no therapeutic benefit, and proceeds with	Bootstrap algorithm can be computationally intensive<br><br>Unadjusted conditional bias can be substantial in some cases	No	Hypothetical example based on the I-SPY 2 Trial

Magnusson and Turnbull (2013)<br><br>https://doi.org/10.1002/sim.5738	the definitive assessment of treatment efficacy among the remaining pooled populations using a group sequential design<br><br>Employs a bootstrap algorithm to obtain point and interval estimates that are adjusted for the selection bias	The double bootstrap adjustment is generally successful at correcting the initial bias (the remaining bias is small relative to the unadjusted bias)<br><br>MSE is usually reduced for adjusted estimators, although the difference is not substantial		
An efficient sequential design of clinical trials<br><br>Cheng and Shen (2013)<br><br>https://doi.org/10.1016/j.jspi.2012.07.015	Proposes an efficient group sequential monitoring rule where at each interim analysis. both efficacy and futility are evaluated through a specified loss structure together with the predicted power.<br><br>For estimation following the proposed design, the authors perform a grid search to find a near unbiased estimator based on Monte Carlo simulations	The obtained estimator has a substantially reduced bias compared with that of the posterior mean. The standard deviation of the proposed estimators are not significantly different from the standard deviation of the posterior mean.	No	None
A flexible method using a parametric bootstrap for reducing bias in adaptive designs with treatment selection<br><br>Pickard and Chang (2014)<br><br>https://doi.org/10.1080/19466315.2014.897251	Describes a novel approach that reduces the bias of the point estimate in treatment selection designs by comparing the observed results to what would be expected when the treatment arms had equal means	The proposed estimator provides a reasonable balance between bias and MSE across several scenarios<br><br>The approach can be applied when the endpoint comes from a normal or binomial distribution, and it can be applied to other distributions as well	No	None

Bootstrap corrections of treatment effect estimates following selection<br><br>Rosenkranz (2014)<br><br>https://doi.org/10.1016/j.csda.2013.08.010	Derives the bootstrap approximation of the bias of the estimators of the maximum effect, the effect of the selected treatment, and the effect of the selected subgroup.	Bootstrap can only partially correct for bias<br><br>Bootstrap method is robust to violations of the assumption of normally distributed data	No	None
Inference for multimarker adaptive enrichment trials<br><br>Simon and Simon (2018)<br><br>https://doi.org/10.1002/sim.7422	Considers a trial design that develops model-based multifeature predictive classifiers as well as optimized cutpoints for continuous biomarkers. A single significance test is performed at the end of the trial of the strong null hypothesis that the expected outcome on the test treatment is no better than control for any of the subset populations of patients accrued in the K stages of the clinical trial<br><br>Uses a parametric bootstrap method to de-bias the estimated treatment effect	Can be computationally intensive<br>Bootstrap bias adjustment was very effective in correcting for this bias regardless of the number of strata	No	None
Point estimation in adaptive enrichment designs<br><br>Kunzmann et al. (2017)	Two-stage adaptive enrichment design where a binary biomarker is applied to select the patient population investigated in stage 2. The outcomes are assumed	EBE and PBE exhibit a higher bias than the MLE in many situations<br><br>CME and hybrid consistently reduce the bias but there is a price to be paid in terms of MSE	No	Illustrative example based on MILLY phase II study in asthma

https://doi.org/10.1002/sim.7412	to be normally distributed with known variance.<br><br>Proposes alternatives to the MLE:<br><br>1. Empirical Bayes Estimator<br>2. Parametric Bootstrap Estimator<br>3. Conditional Moment Estimator<br>4. Hybrid estimator (UMVCUE and MLE or CME)			
Estimation of treatment effects following a sequential trial of multiple treatments<br><br>Whitehead et al. (2020)<br><br>https://doi.org/10.1002/sim.8497	Paper motivated by a trial in which four treatments for sepsis are to be compared, with interim analyses allowing the dropping of treatments or termination of the trial to declare a single winner or to conclude that there is little difference between the treatments that remain.<br><br>Estimation approach based on the method of Rao-Blackwellisation. Analytic approaches to determine such expectations are difficult and specific to the details of the design, and instead “reverse simulations" are conducted to construct replicate realisations of the first interim analysis from the final test statistics.	Reverse simulation approach can be computationally intensive<br><br>The remaining bias is small<br><br>In the two-treatment context, methods based on orderings of the final sample space are just as good for computing point estimates<br><br>The utility of the approach described here is in more complicated designs comparing multiple treatments or with flexible adaptive features, as reverse simulation is based only on the form of the stopping rules implemented and not on their theoretical properties	Yes (SAS)	Hypothetical data based on sepsis trial
